# Tumor-Associated Microglia/Macrophages as a Predictor for Survival in Glioblastoma and Temozolomide-Induced Changes in CXCR2 Signaling with New Resistance Overcoming Strategy by Combination Therapy

**DOI:** 10.3390/ijms222011180

**Published:** 2021-10-16

**Authors:** Ruth M. Urbantat, Claudius Jelgersma, Susan Brandenburg, Melina Nieminen-Kelhä, Irina Kremenetskaia, Julia Zollfrank, Susanne Mueller, Kerstin Rubarth, Arend Koch, Peter Vajkoczy, Gueliz Acker

**Affiliations:** 1 Department of Neurosurgery, Charité–Universitätsmedizin Berlin Corporate Member of Freie Universität Berlin and Humboldt-Universität zu Berlin, Charitéplatz 1, 10117 Berlin, Germany; ruth-maria.urbantat@charite.de (R.M.U.); claudius.jelgersma@charite.de (C.J.); susan.brandenburg@charite.de (S.B.); melina.nieminen@charite.de (M.N.-K.); irina.kremenetskaia@charite.de (I.K.); julia.zollfrank@charite.de (J.Z.); peter.vajkoczy@charite.de (P.V.); 2 Department of Neurology and Experimental Neurology, Charité–Universitätsmedizin Berlin Corporate Member of Freie Universität Berlin and Humboldt-Universität zu Berlin, Charitéplatz 1, 10117 Berlin, Germany; susanne.mueller1@charite.de; 3 NeuroCure Cluster of Excellence and Charité Core Facility 7T Experimental MRIs, Charité–Universitätsmedizin Berlin Corporate Member of Freie Universität Berlin and Humboldt-Universität zu Berlin, Charitéplatz 1, 10117 Berlin, Germany; 4 Experimental and Clinical Research Center, Charité–Universitätsmedizin Berlin Corporate Member of Freie Universität Berlin and Humboldt-Universität zu Berlin, Charitéplatz 1, 10117 Berlin, Germany; kerstin.rubarth@charite.de; 5 Berlin Institute of Health at Charité–Universitätsmedizin Berlin, Charitéplatz 1, 10117 Berlin, Germany; 6 Department of Neuropathology, Charité–Universitätsmedizin Berlin Corporate Member of Freie Universität Berlin and Humboldt-Universität zu Berlin, Charitéplatz 1, 10117 Berlin, Germany; arend.koch@charite.de; 7 Clinician Scientist Program, Berlin Institute of Health at Charité–Universitätsmedizin Berlin, Charitéplatz 1, 10117 Berlin, Germany

**Keywords:** glioblastoma, TAM, CXCR2, CXCL8, IL8, CXCL2, antiangiogenic therapy, combination therapy, temozolomide, SB225002

## Abstract

Tumor recurrence is the main challenge in glioblastoma (GBM) treatment. Gold standard therapy temozolomide (TMZ) is known to induce upregulation of IL8/CXCL2/CXCR2 signaling that promotes tumor progression and angiogenesis. Our aim was to verify the alterations on this signaling pathway in human GBM recurrence and to investigate the impact of TMZ in particular. Furthermore, a combi-therapy of TMZ and CXCR2 antagonization was established to assess the efficacy and tolerability. First, we analyzed 76 matched primary and recurrent GBM samples with regard to various histological aspects with a focus on the role of TMZ treatment and the assessment of predictors of overall survival (OS). Second, the combi-therapy with TMZ and CXCR2-antagonization was evaluated in a syngeneic mouse tumor model with in-depth immunohistological investigations and subsequent gene expression analyses. We observed a significantly decreased infiltration of tumor-associated microglia/macrophages (TAM) in recurrent tumors, while a high TAM infiltration in primary tumors was associated with a reduced OS. Additionally, more patients expressed IL8 in recurrent tumors and TMZ therapy maintained CXCL2 expression. In mice, enhanced anti-tumoral effects were observed after combi-therapy. In conclusion, high TAM infiltration predicts a survival disadvantage, supporting findings of the tumor-promoting phenotype of TAMs. Furthermore, the combination therapy seemed to be promising to overcome CXCR2-mediated resistance.

## 1. Introduction

Glioblastoma (GBM) is the most common malignant brain tumor in adults [1,2]. The standard of care therapy consists of surgical removal of the tumor, followed by concomitant radio-chemotherapy and adjuvant chemotherapy with temozolomide (TMZ) [2,3,4]. Even with this highly aggressive standard of care therapy, a median survival of only 15 months is achieved [5], since GBM is known for a rapid development of resistance to standard therapy with tumor recurrence [6,7,8,9,10]. One of the challenges to overcome therapy resistance is the inter- and intratumoral heterogeneity [10,11,12]. Apart from glioma cells and glioma stem cells, myeloid cells, specifically tumor-associated microglia and macrophages (TAMs), have been shown to act as a driving force of tumor growth and intratumoral diversity [13,14]. Making up for 30–50%, TAMs represent a large proportion of the tumor mass and shape the tumor microenvironment by secreting chemokines, such as CXCL2 and IL8, and growth factors like VEGF and thus contribute to tumor angiogenesis that sustains tumor growth [13,15,16,17,18,19]. As VEGF is overexpressed in GBM [20], and it being one of the most important proangiogenic molecules, anti-VEGF/VEGFR treatment was developed to overcome resistance [19,21,22]. However, promising preclinical findings could only be partially reproduced in clinical studies, and anti-VEGF/VEGFR treatment failed to prolong patients’ overall survival (OS) [21,22,23,24]. One further possible resistance mechanism is the activation of alternative proangiogenic molecules like CXCL2 and IL8 that are also secreted by TAMs [25,26]. These chemokines and their respective receptor CXCR2 play a central role in GBM progression and are associated with a reduced OS [25,26,27,28]. Furthermore, they mediate tumor angiogenesis [25,29] and CXCR2 has been shown to be a driving force on the vascular mimicry (VM) formation in GBM patients, indicating a key role of this signaling pathway in the tumor blood supply [25,30]. 

Importantly, about one-third of TMZ-naïve GBM patients and two-thirds of 17 matched patients overexpressed IL8 in recurrent tumors after TMZ treatment [26,28]. An overexpression of CXCL2 was also observed similar to IL8 in 134 TMZ-naïve GBM patients [26]. Additionally, both IL8 and CXCL2 upregulation was observed in vitro after exposing glioma cells to TMZ, which suggests a potential role of the CXCR2 signaling pathway in the development of resistance against TMZ [31]. Thus, CXCR2 signaling represents a promising additional therapeutical target in GBM treatment to overcome TMZ-induced resistance, as single therapies often fail to target all tumor cells due to GBM heterogeneity [2,3,4]. Recent studies have also shown that combinational approaches exceed the efficacy of sole administration, especially when targeting chemokine signaling axes in addition to the standard of care therapy [32]. In this regard, we recently demonstrated an efficient CXCR2 antagonization with SB225002 (SB) with a significantly reduced tumor volume, decreased vessel density, and reduced TAM infiltration in an in vivo GL261 syngeneic mouse model [6,7,8,9,10]. This treatment approach could be combined with TMZ accordingly. However, only little is known about the impact TMZ might have on the CXCL2, IL8, and CXCR2 expression and on TAMs as a source of these chemokines in GBM during the development of resistance in vivo. Therefore, firstly, we aimed to investigate alterations of TAM infiltration and the CXCR2 signaling pathway in recurrent GBMs in comparison to their matched primary tumors. Secondly, we stratified patients according to the received therapy to assess the effect of TMZ in particular on the CXCR2 signaling pathway and lastly, we investigated the efficacy of TMZ combined with CXCR2 antagonization (SB) compared to TMZ alone in a preclinical syngeneic orthotopic mouse model. 

## 2. Results

### 2.1. Comparison of Matched Primary and Recurrent GBM Tumors

#### 2.1.1. Patient and Tumor Characteristics

To assess general differences between primary and recurrent GBM and TMZ therapy-induced changes, we evaluated a total of 76 tissue samples from 38 GBM patients with matched primary and recurrent tumors (Table 1). The mean age of the patients at diagnosis of the primary tumor was 58.7 years, and 59.6 years at diagnosis of the recurrent tumor. One-third of the patients were female. Median PFS was 9 months (1st quartile: 15.3, 3rd quartile: 5.4) and median OS was 17 months (1st quartile: 29.7, 3rd quartile: 13.5). A total of 18 (47%) patients received more than 4 cycles of TMZ. We then assessed the clinical routine histopathological tumor features that are demonstrated in detail in Table 1. Almost half of the patients (42%) showed a hypermethylated MGMT promotor. MGMT methylation positively correlated with a longer PFS and OS as known from the literature (Supplementary Materials Figure S1a,b) [33,34]. Apart from one patient, all patients carried an IDHwt gene and there were no obvious differences in p53 accumulation in primary and recurrent GBMs (Table 1). With regard to proliferation, most recurrent tumors exerted the same activity as primary tumors. 

#### 2.1.2. TAM Infiltration Is Significantly Reduced in Recurrent Tumors and Very High Infiltration Leads to a Reduced PFS

**Figure 1:**
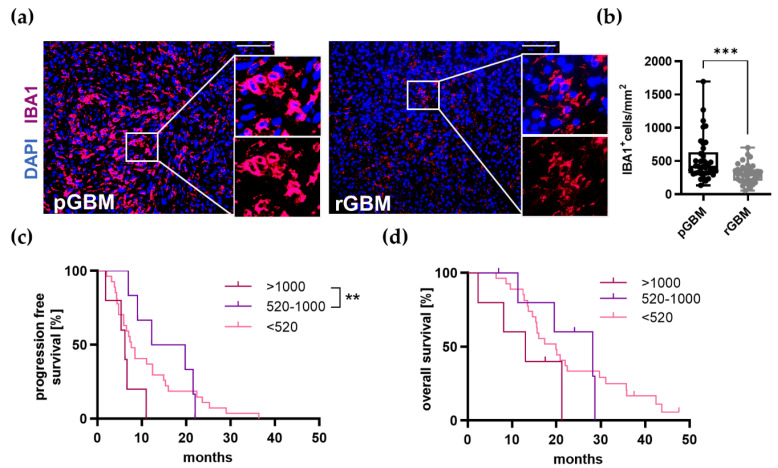
TAM infiltration is significantly reduced in recurrent tumors and a very high infiltration in primary tumors is associated with a shortened PFS. (**a**) Representative immunofluorescence staining of TAMs (IBA1) in magenta and cell nuclei (DAPI) in blue of primary and recurrent tumors of GBM patients, scale bars 100 µm. (**b**) Comparison of the number of TAMs in primary and recurrent tumors showing a significant reduction in recurrent tumors. All primary tumors are depicted in black and recurrent tumors in grey. Box plots depicting mean (shown as “+”), median, 1st and 3rd quartile (shown as the box), and min to max (shown as the whiskers). Mean difference: −217.2; 95% confidence interval −331.1 to −103.3; *n* = 38; *** *p* < 0.001; paired Student’s *t*-test. (**c**,**d**) Patients were then divided into three different groups depending on the amount of infiltrating TAMs in their primary tumors: very high (>1000/mm^2^; *n* = 5), high (520–1000/mm^2^; *n* = 6), and low (<520/mm^2^; *n* = 27) and Kaplan–Meier curves for (**c**) PFS and (**d**) OS were created. Patients within the very high group had a significantly reduced PFS compared to high group. Kaplan–Meier curves with Log-rank test. ** *p* < 0.01.

Since GBMs are infiltrated by a large number of myeloid cells, especially TAMs that make up to 30–50% of the tumor microenvironment [13,14], we first focused on these cells. TAMs are known to exert both pro- [35,36,37] and antitumoral functions [38]. Importantly, they express proangiogenic molecules like VEGF [13,15,16] and CXCL2 [15,17] and thus contribute to maintaining tumor growth. To analyze the amount of TAMs within the tumors, tissue sections were stained for IBA1 (Figure 1a). The number of TAMs was significantly reduced by 41% in recurrent tumors in comparison to primary tumors (*p* = 0.0004; Figure 1b). A subgroup analysis of patients with a very high infiltration (>1000 cells/mm^2^), high infiltration (520–1000 cells/mm^2^), and low infiltration (<520 cells/mm^2^) was then performed. Kaplan–Meier curves revealed that patients with a very high TAM infiltration had a significantly reduced PFS compared to high infiltration (Figure 1c,d; very high vs. high *p* = 0.0079; very high vs. low *p* = 0.1029; high vs. low *p* = 0.6418), while no significant differences could be detected with regard to the OS within the categorized TAM infiltration groups. Interestingly, the median survival of patients within the very high (6.25 months) and the low infiltration group (7.63 months) was less than in the high infiltration group (16.01 months); however, this did not reach the level of significance (OS: very high vs. high *p* = 0.1081; very high vs. low *p* = 0.1538; high vs. low *p* = 0.9790).

#### 2.1.3. Comparable Angiogenic Activity in Primary and Recurrent Tumors

**Figure 2:**
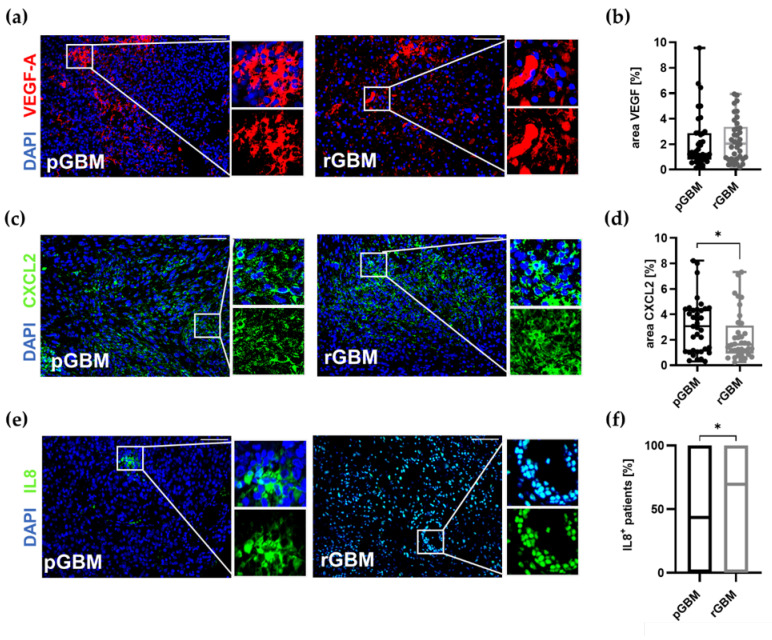

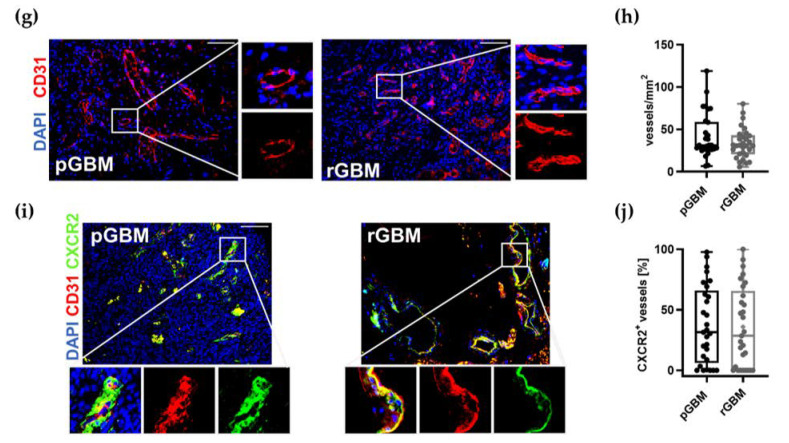
The expression of proangiogenic molecules is distinctly changed in recurrent GBM tumors. Representative images of FFPE tissue sections of primary and recurrent tumors stained for (**a**) VEGF (red), (**c**) CXCL2 (green), (**e**) IL8 (green), (**g**) blood vessels (CD31; red), (**i**) CXCR2 (green), and cell nuclei (DAPI; blue); scale bars: 100 µm. (**b,d**) Graphs show calculation of the stained area of VEGF (**b**) and CXCL2 (**d**) as a percentage in relation to the total analyzed tumor area (*n* = 38). (**f**) The percentage of patients expressing IL8 in primary and recurrent tumors was assessed (*n* = 38). (**h**,**j**) Graphs depict vessels/mm^2^ and the percentage of CXCR2^+^ vessels (*n* = 28–30). All primary tumors are depicted in black and recurrent tumors in grey. Box plots depicting mean (shown as “+”), median, 1st and 3rd quartile (shown as the box), and min to max (shown as the whiskers). * *p* < 0.05. Paired Student’s *t*-test; box plots depicting mean (shown as “+”) and median ± standard deviation.

Besides high TAM infiltration, GBM is also characterized by a strong angiogenic activity [19]. Thus, we investigated the expression of VEGF (Figure 2a,b) and the alternative proangiogenic factors CXCL2 (Figure 2c,d) and IL8 (Figure 2e,f) amongst the matched tumor samples. While there were no changes in the expression of VEGF, CXCL2 expression was significantly reduced in recurrent GBMs (*p* = 0.0280) compared to primary tumors. Interestingly, all patients expressed CXCL2 in the primary tumors while IL8 was only detected in 43% (*n* = 16) of primary GBMs. In recurrent tumors, IL8 expression was significantly increased to 67.5% (*n* = 25) (*p* = 0.0438). To further evaluate the influence of alterations in recurrent tumor vascularization, all tumor sections were stained for the endothelial cell marker CD31. The vessel count in recurrent tumors did not differ from primary tumors (Figure 2g,h; pGBM: 41/mm^2^ vs. rGBM: 34/mm^2^). As CXCR2 is preferentially expressed by endothelial cells, co-staining of CD31 and CXCR2 was carried out (Figure 2i). However, no differences in the percentage of CXCR2-positive blood vessels could be detected between primary and recurrent GBM tumor samples (Figure 2j; pGBM: 36% vs. rGBM: 40%). 

In summary, recurrent GBM tumors were different to their matched primary counterpart with regard to TAM infiltration and the expression of the alternative proangiogenic molecules CXCL2 and IL8, while VEGF and the tumor vascularization remained comparable.

#### 2.1.4. TAMs Serve as a Predictor for a Reduced OS

To further assess the impact of the examined parameters on patient survival, an explorative cox regression analysis was carried out. The influence of the TAM infiltration, the expression of VEGF, CXCL2, and IL8 as well as vascular parameters, such as the vessel density, vessel area, including CXCR2^+^ vessels and their impact on the OS, and the PFS, were examined. In order to evaluate the effect of temozolomide on patient survival, the received temozolomide cycles were included in this analysis as an independent parameter, since the number of the TMZ cycles was not uniform in our patient cohort. Furthermore, MGMT hypermethylation is associated with a prolonged OS as described in the literature and confirmed in our cohort [33,34] (Supplementary Materials Figure S1a,b). Therefore, the MGMT status was included as well. 

The univariable analysis of the TAM infiltration showed that it is a positive predictor for a significantly reduced OS (*p* = 0.02). The multiple analysis then confirmed that TAM infiltration is an independent positive predictor (*p* = 0.02) and that the CXCR2^+^ vessel area is an independent negative predictor of overall survival in GBM patients as well (*p* = 0.04) (Table 2). Therefore, patients with a greater CXCR2^+^ vessel area may survive longer (Table 2). Subsequently, we performed a subgroup analysis that only included patients with CXCR2^+^ vessels (Supplementary Materials Table S2). Here, multiple analysis of TAM infiltration, received TMZ cycles, and CXCR2^+^ vessel area did not confirm the CXCR2^+^ vessel area as an independent negative predictor of OS (Supplementary Materials Table S2). The amount of received TMZ cycles and the hypermethylation of MGMT did not predict the OS in this study (Table 2). However, the number of received TMZ cycles served as a negative predictor of recurrence (*p* = 0.02). Consequently, patients receiving more TMZ cycles had a later recurrence in our cohort, confirming the protective role of TMZ treatment against tumor progression (Table 3). The only other predictor for a prolonged PFS was MGMT methylation. Both factors remained significant in the multiple analyses (Table 3).

### 2.2. Potential Effects of TMZ Treatment on the Tumor Microenvironment

#### Expression of Proangiogenic Molecules and Vascularization in GBM Patients Is Mostly Not Affected by TMZ Therapy

**Figure 3:**
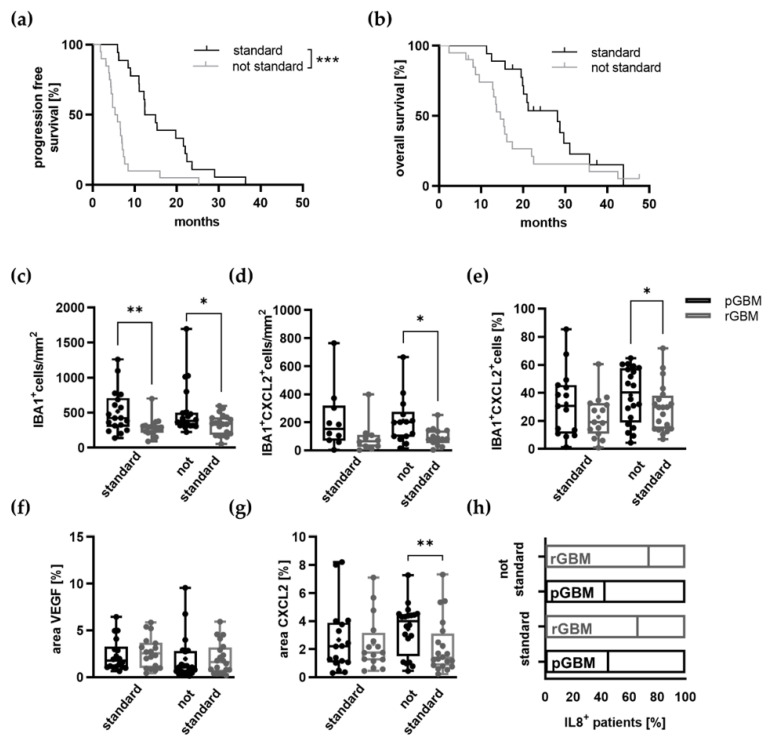

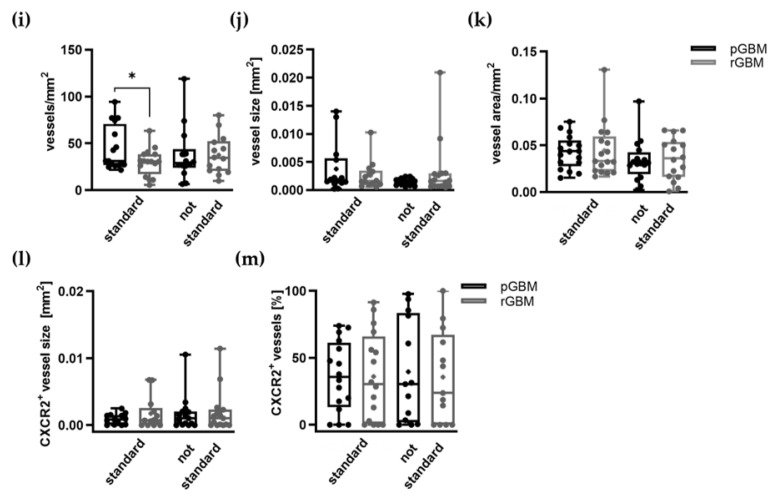
Influence of TMZ on TAM infiltration, tumor vascularization, and expression of proangiogenic molecules in matched primary and recurrent GBMs. (**a**,**b**) Kaplan–Meier curves for (**a**) progression-free (PFS) and (**b**) overall survival (OS) of the subgroup analysis comparing the standard with the not standard group. (**c**–**m**) Graphs show the calculation of the stained area of TAMs (IBA1) (**c**–**e**), proangiogenic molecules VEGF (**f**), CXCL2 (**g**), IL8 (**h**) tumor vessels (CD31) (**i**–**k**), and CXCR2^+^ vessels (**l**,**m**) on FFPE tissue sections of primary and recurrent tumors of all patients. (**c**) Graph depicts the amount of TAMs in primary and recurrent tumors of both groups, which were significantly reduced in recurrent tumors (standard: mean difference: 246.7; 95% confidence interval −400.5 to −92.87; not standard: mean difference: −192.2; 95% confidence interval −370.0 to −14.40). (**d,e**) Graphs depict CXCL2^+^ TAMs in primary and recurrent tumors of both groups. (**f**–**h**) Graphs show calculation of the stained area of VEGF (**f**) and CXCL2 (**g**) (*n* = 38; standard: *n* = 18; not standard: *n* = 20). (**h**) The number of IL8^+^ patients in primary and recurrent tumors was calculated (*n* = 38; standard: *n* = 18; not standard: *n* = 20). (**i**–**k**) Graphs depict vessels/mm^2^ (**i**), the vessel area/vessel number (**j**), and vessel area/mm^2^ (**k**) (*n* = 30; standard: *n* = 15; not standard: *n* = 15). (**l**,**m**). The area of CXCR2^+^ vessels/CXCR2^+^ vessel number in (**l**) and the percentage of CXCR2^+^ vessels in relation to all vessels (**m**) was calculated (*n* = 28; standard: *n* = 13; not standard: *n* = 15). * *p* < 0.05, ** *p* < 0.01, *** *p* < 0.001. (**c**–**m**) paired Student’s *t*-test; box plots depicting mean (shown as “+”), median, 1st and 3rd quartile (shown as the box) and min to max (shown as the whiskers).

TMZ is known to have a high impact on prolonging patients’ lives [2]. This was also confirmed by our study as a higher number of received TMZ cycles predicted a longer progression-free survival. Furthermore, we observed significant differences between primary and recurrent tumors with regard to TAM infiltration and expression of the proangiogenic factors CXCL2 and IL8. It has been shown that gene expression of TAMs differed between matched primary and recurrent tumors (*n* = 19); however, it is not known whether TAM reduction and alterations in angiogenic pathways in recurrent tumors are associated with the duration of TMZ treatment [39]. Therefore, we then aimed to analyze the role of TMZ on these alterations in particular. In our cohort, all patients underwent a primary tumor resection followed by radio-chemotherapy (*n* = 32; 84%) or in a few cases sole radiotherapy (*n* = 4; 10%). However, as the de facto applied treatment was not uniform amongst the patients regarding the number of TMZ cycles, we divided the patient cohort into two groups as described in the methods in order to assess the effects of TMZ therapy, fulfilling almost the standard adjuvant regimen (standard: TMZ^≥4^) in comparison to none or only few applied cycles (not standard: TMZ^≤3^). Relevant clinical comparison of both groups is presented in Supplementary Materials Table S1. To evaluate whether the two groups were different at the point of diagnosis, patient age, gender, and primary tumor characteristics including MGMT status and IDH and p53 accumulation were assessed and showed no significant differences (Supplementary Materials Table S1). Kaplan–Meier curves were generated and showed a distinct prolonged PFS (standard: 13.7 vs. 5.6 months; *p* = 0.0002) of the standard group (Figure 3a). However, the overall survival was not significantly different (standard: 28.2 vs. not standard: 14.6 months; *p* = 0.0525) (Figure 3b). We then evaluated the impact of TMZ therapy on the infiltration of TAMs, the expression of proangiogenic molecules, and the vasculature in primary and recurrent GBMs and compared both groups. First of all, the infiltration of TAMs remained significantly lower in recurrent tumors compared to primary tumors regardless of the treatment groups (standard: *p* = 0.0037; not standard: *p* = 0.0356; Figure 3c). As TAMs serve as a source of the proangiogenic molecule CXCL2, TAMs that showed expression of CXCL2 were counted in particular. In the recurrent tumors of both groups, the amount of CXCL2^+^ TAMs per mm^2^ and the percentage of CXCL2-expressing TAMs were reduced. However, this reduction only reached the level of significance in the not standard group (not standard: CXCL2^+^ TAMs per mm^2^: 46% decrease in recurrent tumors, *p* = 0.0147, Figure 3d; percentage of CXCL2^+^ TAMs: 23% decrease in recurrent tumors, *p* = 0.0383; Figure 3e and Supplementary Materials Figure S2a,b).

We then evaluated differences regarding IL8 and CXCL2 expression in recurrent tumors of both groups and also the standard proangiogenic molecule VEGF. IL8 expression increased in recurrent tumors independent of the therapeutic regimen (standard: 38% increase, *p* = 0.2686; not standard: 85% increase, *p* = 0.05527) (Figure 3h). The area of CXCL2 expression was only significantly reduced in recurrent tumors of patients in the not standard group (standard: 10.1% decrease; not standard: 36.5% decrease; *p* = 0.0055) (Figure 3g). Interestingly, 50% of patients within the standard group (TMZ^≥4^) showed an upregulation of CXCL2 in the recurrent tumors. While IL8 and CXCL2 expression was changed in recurrent tumor, there was no significant difference regarding the expression of VEGF (Figure 3f). Angiogenic molecules promote tumor vascularization. Thus, vessel structures of primary and recurrent tumors in both groups were analyzed in addition to the angiogenic pathways. The vessel count, vessel size, vessel area as well as CXCR2^+^ vessels and area/CXCR2^+^ vessel were evaluated. The amount of vessels per mm^2^ in the recurrent tumors of the standard group was markedly decreased (standard: 28.7% decrease in rGBM; *p* = 0.0369: not standard: 0.5% decrease in rGBM). All other parameters were not differently affected by the different therapeutic regimes (Figure 3i–m). 

### 2.3. In Vivo Assessment of Combination Therapy with TMZ and SB

The reduced TAM infiltration in recurrent GBM underlines the relevant morphological differences between primary and recurrent tumors in line with the changed gene profile of recurrent GBM tumors, as has already been shown [39]. These data reflect the need for different therapeutic approaches in recurrent tumors. As our data confirmed the crucial role of TAMs in tumor progression of primary tumors and their association with a reduced overall survival in GBM patients, TAMs are a suitable target for the initial GBM treatment. In our previous study, we showed that the local treatment by CXCR2 antagonist SB225002 (SB) significantly reduced the TAM infiltration in an orthotopic glioma mouse model in vivo [40]. Furthermore, in our patient cohort, CXCL2 was not significantly reduced in recurrent tumors after therapy according to Stupp’s protocol in contrast to recurrent tumors of patients who received no or less temozolomide. This finding matched the previously described TMZ-induced changes in the chemokine network, which could be identified as a mechanism to the development of therapy resistance [28,31]. Even if only a few effects of TMZ application were observed on CXCR2 signaling in matched patient samples, preclinical models showed the efficacy of combined approaches consisting of standard therapy and additionally targeting chemokine signaling axes [32]. Therefore, combining SB additionally with TMZ poses a promising therapeutic approach. To validate the treatment tolerability and the efficacy of combined therapy of TMZ and SB, a GBM mouse model with GL261 tumor cells was used.

#### 2.3.1. No Adverse Effects by Additional CXCR2 Antagonization with TMZ In Vivo 

The combination of SB and TMZ was well tolerated during the treatment period of seven days. Animals showed no signs of additional distress and did not develop any side effects. Additionally, the body weight was not influenced by therapy. During preparation for perfusion, slightly irritated intestines were observed, most likely caused by intraperitoneal TMZ administration as this was also the case for the sole TMZ group. 

#### 2.3.2. Combination Therapy with TMZ and SB Reduces Tumor Volume and Proliferation in a Syngeneic Orthotopic GBM Mouse Model

**Figure 4:**
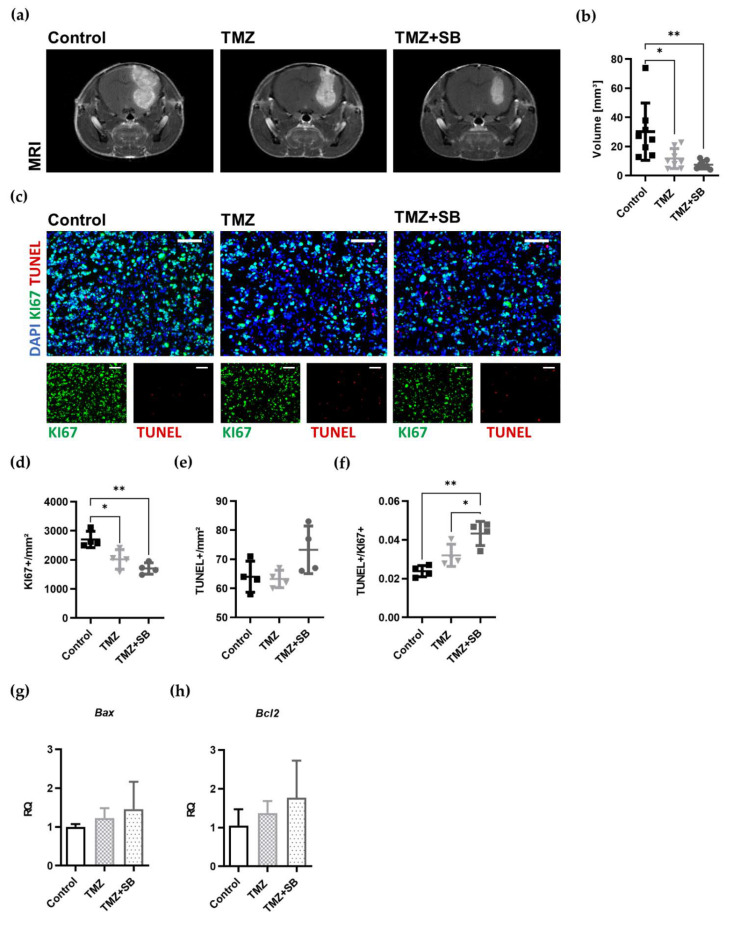
Post treatment MRIs of tumor volume and therapy-induced effects on proliferation and apoptosis. (**a**) Representative T1w MRI images after contrast agent administration are showing a therapy-induced reduction of tumor volume after the treatment with TMZ and TMZ + SB. (**b**) Quantitative tumor volumetry showed a significantly reduced tumor volume by 75% in the combination group (TMZ + SB) whereas TMZ alone reduced tumor volume by 61% (*n* = 7–8; * *p* = 0.0224; ** *p* = 0.0062). (**c**) Representative immunofluorescence staining of each group showing a reduction of proliferation in the TMZ and TMZ + SB group while apoptosis seemed to be unaffected (Ki67: green, TUNEL: red, scale bar = 100 µm). (**d**) The detailed analysis of proliferation showed significant impairment in both treatment groups. The proliferation was reduced by 37% in the combination group and 25% in the TMZ group (*n* = 4; * *p* = 0.0205; ** *p* = 0.002; TMZ vs. TMZ + SB, *p* = 0.4416). (**e**) However, apoptosis remained unaltered (*n* = 4; Control vs. TMZ, *p* = >0.9999; Control vs. TMZ + SB, *p* = 0.1616; TMZ vs. TMZ + SB, *p* = 0.1204). (**f**) If the ratio of apoptosis and proliferation was calculated, a significant shift towards apoptosis could be detected only in the combination group of TMZ + SB225002 (*n* = 4; * *p* = 0.0388; ** *p* = 0.0014). (**g**,**h**) Apoptosis-regulating molecules *Bax* (proapoptotic) and *Bcl2* (antiapoptotic) were unchanged in qRT-PCR analysis (*n* = 3–4). (**d**–**f**) Graphs depict individual values with additional mean ± standard deviation; bar graphs (**g**,**h**) showing mean ± standard deviation. One-way ANOVA with Bonferroni correction was performed in all analyses.

Tumor growth was assessed by MRI before starting the treatment on day 14. After seven days, tumor volume was again assessed by MRI and histological analysis were conducted. In the combined treatment group, a tumor volume reduction of 75% was achieved compared to the control group (*p* = 0.0062, *n* = 7–8), whereas TMZ alone led to a reduction of 61% (*p* = 0.0224, *n* = 7–8; Figure 4a,b). The additional effect through adding SB to TMZ was 36% compared to sole TMZ treatment; however, this did not reach a significant level. 

To investigate the underlying mechanisms of tumor volume reduction in detail, the effects of TMZ and SB on proliferation and apoptosis were investigated. It has already been shown that both TMZ and SB have an inhibitory influence on the proliferation of tumor cells [4,40,41,42,43]. It was therefore interesting to see whether the effects of both substances could potentiate one another. In this study, a significantly impaired proliferation could be observed in both treatment groups compared to the untreated control (TMZ group: by 25%; *p* = 0.0205, *n* = 4; TMZ + SB: by 37%; *p* = 0.002, *n* = 4; Figure 4c,d). Interestingly, apoptosis was unaltered in the setting used in this study (Figure 4c,e). However, when the ratio of apoptotic and proliferating tumor cells was calculated, a shift towards apoptosis could be detected following combined treatment (compared to control: by 82%; *p* = 0.0014, compared to TMZ alone: by 35%; *p* = 0.0388; *n* = 4; Figure 4f). 

For further verification of these findings, gene expression analysis of *Bax* (proapoptotic) and *Bcl2* (antiapoptotic), both regulators of apoptosis, were conducted [44,45]. Within a treatment period of seven days, *Bax* and *Bcl2* gene expression were not changed by either of the therapies (Figure 4g,h). 

Taken together, the antitumoral effect of combinational treatment on tumor volume reduction and proliferation exceeded the effect of sole TMZ. 

#### 2.3.3. Combination Therapy with TMZ and SB Tends to Diminish Total Tumor Vascularization While Infiltration of TAMs Was Unaltered 

**Figure 5:**
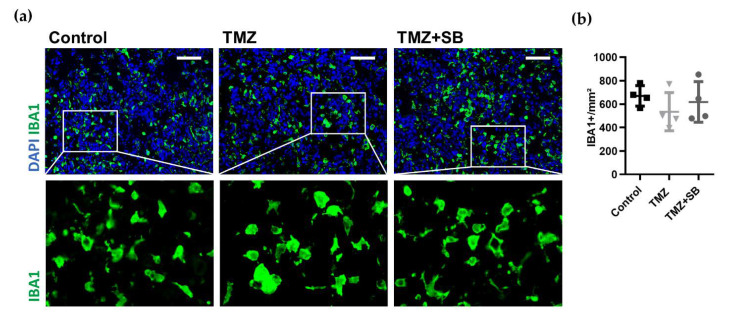

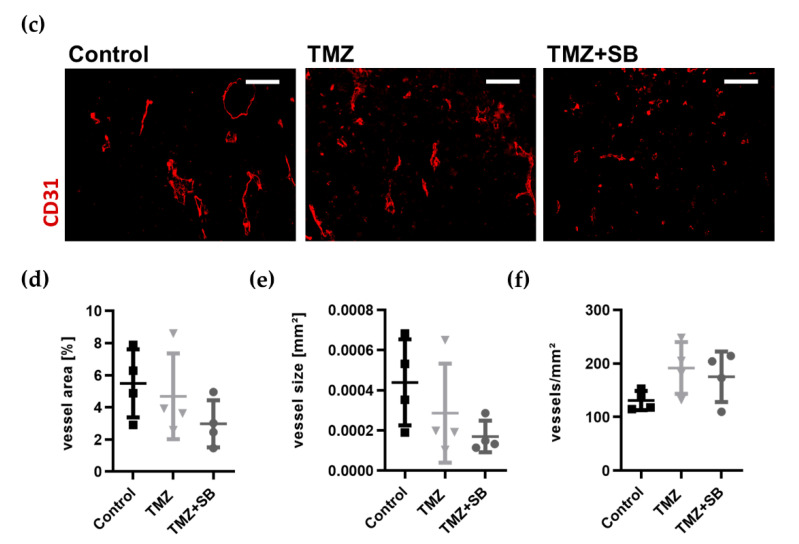
Therapy-induced effects on TAM accumulation and tumor vascularization. (**a**) Representative immunofluorescence staining showing no differences in TAM accumulation between groups (DAPI: blue, IBA1: green, scale bar = 100 µm). Additionally, no obvious changes in TAM morphology by therapy were observed as indicated in the magnified images. (**b**) The quantitative analysis of TAM accumulation showed comparable levels between all groups. (**c**) Representative immunofluorescence staining showing a tendency of diminished tumor vascularization (CD31: red, scale bar = 100 µm). (**d**–**f**) Quantitative comparison of vessel parameters showed reduced vessel area (calculated as a percentage in relation to the total analyzed tumor area; *n* = 4; Control vs. TMZ, *p* ≥ 0.9999; Control vs. TMZ + SB, *p* = 0.3951; TMZ vs. TMZ + SB, *p* = 0.8661) and average vessel size (calculated as total vessel area/vessel number; *n* = 4; Control vs. TMZ, *p* = 0.8802; Control vs. TMZ + SB, *p* = 0.2449; TMZ vs. TMZ + SB, *p* ≥ 0.9999) which was most markedly in the combination group. Vessel density was unchanged in all groups (*n* = 4; Control vs. TMZ, *p* = 0.1841; Control vs. TMZ + SB, *p* = 0.4623; TMZ vs. TMZ + SB, *p* ≥ 0.9999). Graphs depict individual values with additional mean ± standard deviation. One-way ANOVA with Bonferroni correction was performed in all analyses.

Our data supports the pro-tumoral role of TAMs in GBM pathogenesis as a predictor for reduced overall survival as demonstrated in the previous section and furthermore our group has recently reported a decrease in TAMs after sole SB treatment [40]. Based on these findings, it was of major interest to analyze if TAMs play a role in the additional anti-tumoral effect of the combination therapy in vivo. However, accumulation of TAMs was unaltered in vivo amongst all groups within the short observation period (Figure 5a,b).

CXCR2 signaling is involved in central angiogenic cascades and the selective CXCR2 antagonist SB showed antiangiogenic potential in recent studies [25,26,40]. Therefore, we aimed to investigate tumor vascularization in detail. The question was whether adding CXCR2 antagonization to TMZ can enhance antiangiogenic efficacy of the therapy. Overall, we observed an incipient decrease of tumor vascularization, which was most distinctive in the combined administration of TMZ + SB (Figure 5c–f). In comparison with sole TMZ and combined treatment, the vessel area and average vessel size were reduced by 36.5% and 40.5%, respectively (Figure 5d,e). Nevertheless, the threshold of significance was not crossed. No difference between TMZ and TMZ + SB could be detected with regard to the vessel count (Figure 5f).

Overall, we observed a tendency for diminished vessel area and vessel size without alterations in TAM accumulation after combinational treatment in comparison to sole TMZ.

#### 2.3.4. CXCR2/CXCL2 Are Upregulated in Murine Tumor Tissue and Decreased by Therapy without Affecting Alternative Signaling Pathways

**Figure 6:**
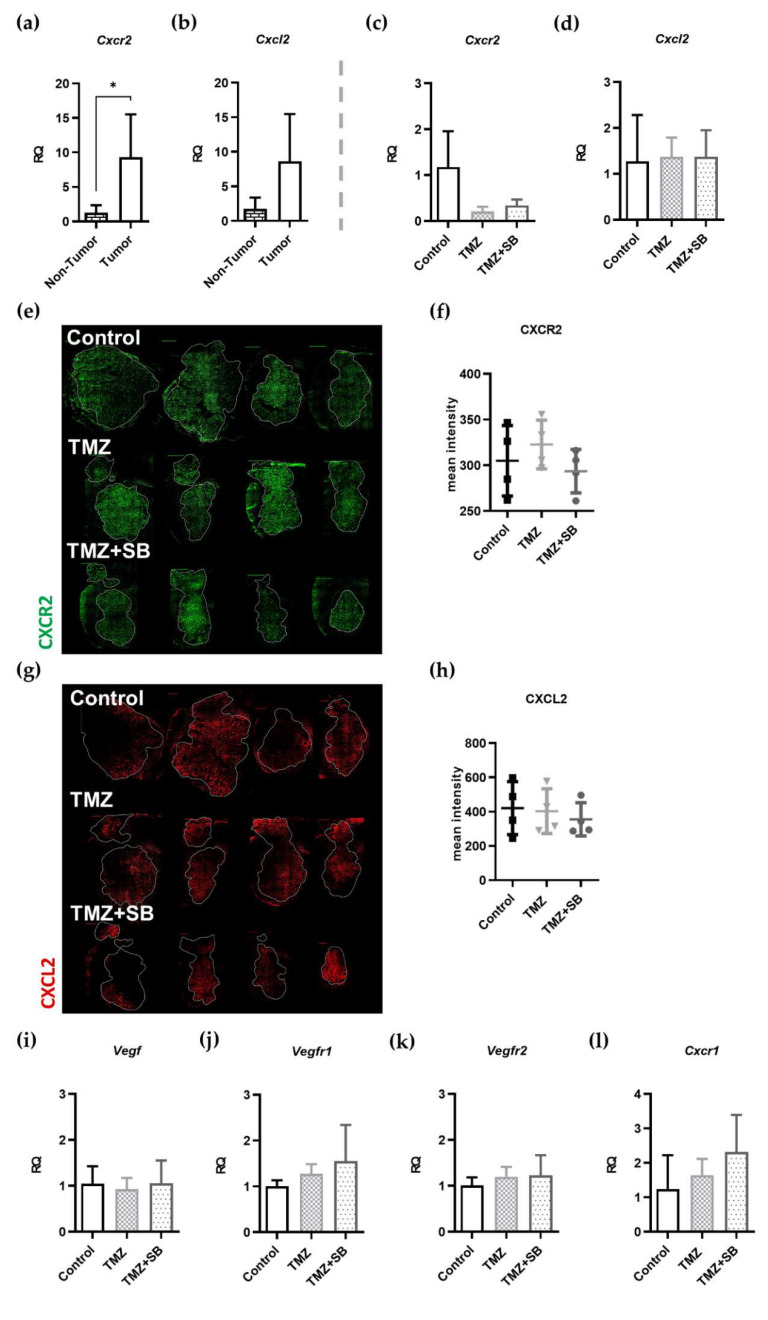
Upregulation of CXCL2/CXCR2 signaling in tumor tissue and the influence of therapy on CXCR2 signaling and alternative pathways. (**a**) Gene expression of *Cxcr2* was significantly upregulated (7-fold) in tumor tissue of GL261 mouse GBMs compared to contralateral brain parenchyma (*n* = 4; *p* = 0.0434). (**b**) Additionally, *Cxcl2* was upregulated 5-fold but did not a significant level in this study (*n* = 4; *p* = 0.0977). (**c**) The investigation of *Cxcr2* showed a therapy-induced decrease of gene expression under TMZ and TMZ + SB treatment (*n* = 3–4, Control vs. TMZ, *p* = 0.0693; Control vs. TMZ + SB *p* = 0.1637) but without reaching statistical significance. (**d**) However, *Cxcl2* remained completely unchanged (*n* = 3–4). (**e**–**h**) Representative immunofluorescence staining of CXCR2 and CXCL2 on the protein level showed no differences or group-specific distribution patterns amongst all groups (CXCR2: green, CXCL2: red). The measurement of the mean intensity of immunofluorescence images of both molecules showed no differences of protein expression. All images were captured with the same exposure time under comparable conditions (*n* = 4). (**i**–**k**) qRT-PCR analysis of the classic angiogenic pathway with *Vegf* and its receptors *Vegfr1* and *Vegfr2* showed no compensatory upregulation during therapy with TMZ or TMZ + SB (*n* = 3–4). (**l**) Gene expression of *Cxcr1*, which has similar downstream effects as *Cxcr2*, was also not altered significantly (*n* = 3–4). (**a**–**d**,**i**–**l**) Bar graphs showing mean ± standard deviation; (**f**,**h**) graphs depict individual values with additional mean ± standard deviation; one-way ANOVA with Bonferroni correction was performed in all analyses.

An important requirement for investigating the effect of adding the CXCR2 antagonist SB to the TMZ standard therapy was the upregulation of the CXCR2 signaling pathway in our tumors in comparison to healthy brain tissue. Based on qRT-PCR analysis, upregulation of *Cxcr2* and *Cxcl2* were detected in untreated tumor tissue compared to contralateral non-tumor tissue (Figure 6a,b). While *Cxcr2* was significantly upregulated ≈ 7-fold (*p* = 0.0434, *n* = 4; Figure 6a), *Cxcl2* upregulation was measured as 5-fold with a high variation of individual values that did not reach the level of significance (*n* = 4, *p* = 0.0977; Figure 6b). Interestingly, both therapies induced a decrease of *Cxcr2* (Control vs. TMZ: 5.6-fold; Control vs. TMZ + SB: 3.5-fold; *n* = 3–4; Figure 6c), whereas *Cxcl2* expression remained unaffected (Figure 6d). Furthermore, the expression of CXCR2 and CXCL2 were verified on the protein level via immunohistological intensity measurements. No differences could be seen in both therapy groups compared to the control (Figure 6e–h). Importantly, molecule staining showed no group-specific distribution pattern amongst all groups (Figure 6e,g).

To unravel other therapy-induced changes or compensatory mechanisms that could be a reason for the only slightly decreased tumor vascularization, further qRT-PCR analyses were conducted, with a focus on classic proangiogenic molecules, such as *Vegf* with its receptors *Vegfr1* and *Vegfr2* as well as *Cxcr1*. It is known that the VEGF pathway is also upregulated in GBM pathogenesis and correlates with tumor vascularization [19,46]. Furthermore, CXCR1 serves as an alternative binding site for CXCR2 ligands, including IL8 and CXCL2, leading to similar effects to CXCR2 after activation [47,48,49,50]. After the treatment period of seven days, no altered gene expression levels of either the *Vegf* pathway nor *Cxcr1* was detected (Figure 6i–l). 

In summary, it can be said that CXCR2 was significantly upregulated in the GL261 GBM tumor model compared to healthy tissue, with a decrease after each therapy. Importantly, alternative signaling pathways were not altered in vivo. 

## 3. Discussion

Our analysis of 76 matched primary and recurrent GBM samples underlined important morphological differences between primary and recurrent GBM, with a higher infiltration of TAMs in the primary tumors. Our data revealed that the infiltration of TAMs in primary tumors served as a negative predictor for patients´ OS. Only minor possibly TMZ-induced differences regarding the important vasculogenic and tumorigenic pathways, VEGF and CXCR2, in GBM patients could be detected. However, CXCL2 expression was significantly lower in recurrent tumors of the not standard (TMZ^≤3^) group, while IL8 expression rose from 45% (standard) and 42% (not standard) in primary tumors to 61% and 74%, respectively, in recurrent tumors. The combination of TMZ with CXCR2-antagonization represented a new promising treatment approach in GBM based on the previous studies [25,26,28,32,40,51,52,53]. Here, we demonstrated a well-tolerated therapy regimen with an enhanced reduction of tumor volume and proliferation by adding SB to the TMZ treatment in a syngeneic orthotopic mouse model. 

### 3.1. Comparison of Matched Primary and Recurrent GBM Tumor Characteristics 

To assess whether our matched GBM patient cohort was representative for GBM patients, clinical characteristics and neuropathological routine diagnostics were analyzed. The age at diagnosis and the gender distribution in our study cohort was comparable to previously published data [2,54]. In this study, patients within the standard group had a median OS of 28.6 months (1st quartile: 31.1, 3rd quartile: 19.9) compared to 14.6 months (1st quartile: 22.0, 3rd quartile: 9.6) within the not standard group. Thus, this exceeded the expected median OS for both groups, when compared to previous studies [2,54]. According to the published data, only 20–30% of GBM patients undergo a second surgical treatment [55], depending on their age and clinical performance status [56]. As the development of tumor recurrence and surgical treatment for both the primary and recurrent tumors were the main inclusion criteria of this study, the patient selection could be biased, leading to the positive influence on the OS. Other characteristics that are associated with a prolonged survival are IDH-1/2 mutations and MGMT hypermethylation [57]. However, IDH was only mutant in one patient. Previous studies have shown that the MGMT promoter is hypermethylated in 40–60% of GBM patients, which coincides with our findings [58]. We could confirm that MGMT methylation positively correlates with PFS and OS in our patient cohort as previously shown by Radke et al. [34]. Nevertheless, in contrast to their findings, we did not identify high MGMT methylation as a predictor of survival, which could be due to the fact that they divided patients into groups according to the MGMT methylation level [34].

### 3.2. TAMs Serve as a Negative Predictor of OS in GBM

It is known that the amount of TAMs correlates positively with tumor grade and malignancy [59,60]. For the first time, we identified TAMs as a negative predictor of OS in our GBM patient cohort. This is an important finding as several studies suggest that TAMs exert pro- or anti-tumoral functions depending on their polarization in GBM (M1 = proinflammatory, antitumoral; M2 = immunosuppressive, protumoral) [13,61,62,63]. Furthermore, emerging evidence indicates that TAMs acquire different phenotypes dependent on the tumor microenvironment [18,62,63,64,65]. However, within the past years, several research groups have indicated that TAM populations within GBM showed heterogenous gene expression, which cannot be assigned to the classical M1 or M2 phenotype, but rather to a specific subtype of protumoral TAMs [18,63,64]. Our findings of TAMs being a negative predictor of survival support the pro-tumoral phenotype of these myeloid cells in primary GBM as previously described by others [35,36,37]. Importantly, a very high infiltration led to a shorter PFS in our cohort, while the intermediate (high) group had the best median overall survival, suggesting a required balanced number of TAMs for tumor control. To clarify this phenomenon, further investigations on TAM gene expression are warranted. 

Furthermore, infiltration of TAMs was significantly reduced in the recurrent tumors compared to their matched primary tumors. A recent study by Fu et al. demonstrated that the proportion of TAMs in relation to the total amount of immune cells was decreased in three recurrent GBM samples [66]. As they calculated the proportional amount and only investigated a small number of patients without using matched primary and recurrent tumors, our study is the first to report that the amount of TAMs is significantly reduced in recurrent GBMs in a cohort of 38 matched tumors. Importantly, Hudson et al. demonstrated that TAMs in primary tumors differ from their counterparts in recurrent tumors in a matched patient cohort (*n* = 19) [39]. This, alongside our findings, needs to be taken in consideration regarding future immune-therapeutic approaches. 

To distinguish between therapy-induced changes, especially with regard to TMZ cycles, we performed subgroup analyses with two groups (standard, not standard) based on the received amount of chemotherapy. Interestingly, TAM infiltration was reduced in recurrent tumors independent of the received therapy. Despite the reduced TAM infiltration, the percentage of patients expressing IL8 rose by more than 20% in the recurrent tumors compared to the primary tumors in both treatment groups. Furthermore, CXCL2 expression was significantly reduced in patients of the not standard group in the recurrent tumors whereas CXCL2 expression in patients receiving temozolomide according to the Stupp protocol was comparable to primary tumors. Additionally, the amount of CXCL2-expressing TAMs was significantly reduced in the recurrent tumors of the not standard group, which may account for the reduced amount of CXCL2. These findings implicate a possible increase in CXCL2 expression after ≥4 cycles of TMZ, if the decrease in CXCL2-expressing TAMS and CXCL2 itself in the less or no TMZ group (TMZ^≤3^) could be considered representative for the natural course of tumor recurrence. This interpretation would coincide with the previously described upregulation of CXCL2 and IL8 after TMZ treatment in vitro and in GBM patients [28,31]. However, as we could only include a few patients without any TMZ treatment (*n* = 4), this can only be assumed and needs to be proven in future studies. As TMZ is part of the standard treatment, patients who are not treated with it are rare and mostly >70 years. This age group is often treated according to the Nordic glioma protocol [67]. Furthermore, it would be unethical to withhold this part of the well-established therapy from GBM patients, especially because median survival with the standard of care therapy is still only 15 months [2].

### 3.3. The Influence of TMZ on Central Chemokines in GBM

Most studies on CXCR2 signaling in GBM have focused on IL8 rather than CXCL2 [25,28,50,68]. In this regard, it is important to mention that IL8 is not expressed in immunocompetent murine models and CXCL2 has been identified as the respective IL8 homologue [69]. We showed that CXCL2 is expressed by all patients in primary GBM tumors while IL8 was only detected in 43% of primary tumors but in 70% of recurrent tumors. This finding coincides with a recently published study by Hasan et al. [28]. Here, the authors showed that IL8 was elevated in 65% of 17 matched recurrent GBMs [28]. On the other hand, Bruyère et al. showed that CXCL2 and CXCR2 were expressed by three out of four patient-derived GBM cell lines while IL8 was expressed by all of them on the mRNA expression level [31]. These findings raise the question of whether CXCL2 and IL8 really exert the exact same functions in GBM patients, or whether they act complementary to each other. This is of major interest for future studies to understand this signaling pathway better. Nevertheless, the upregulation of the CXCR2 axis has been shown to be associated with a reduced overall survival in GBM patients, which further underlines the importance of this signaling pathway regardless of the binding chemokine [25,26]. Therefore, CXCL2 and IL8 seem be important to fight GBM recurrence. Thus, blocking their mutual receptor CXCR2 represents a promising target to establish new therapeutic approaches.

### 3.4. Combination Therapy Leads to Superior Antitumoral Effects Compared to Sole TMZ In Vivo

Combination treatments can outperform any single approach by combining synergistic effects while lowering the required concentration of each active ingredient to reduce side effects [51]. As recently shown by our group, intrathecal use of SB reduced tumor volume in the same syngeneic orthotopic mouse model by 47% [40] but failed to cure the disease. This again underlines the need for a combined approach. As TAM infiltration affected primary tumor progression in our study, and CXCR2 antagonization has been shown to reduce TAM accumulation [40], a combination strategy utilizing a CXCR2 antagonist and TMZ seemed appealing. In this study, the combined application of SB and TMZ led to a tumor reduction of 75% within a very homogeneous group while sole TMZ application reduced tumor volume by 61%. This increase in volume reduction could be best explained by blockage of CXCR2 signaling in addition to TMZ treatment. Even though there was no significant difference between the TMZ only and the TMZ + SB group in proliferation and apoptosis, the ratio of apoptosis to proliferation was only significantly shifted in the combined treatment group compared to the control and sole TMZ group. 

In our previous studies, we reported a reduced vessel density in vivo and diminished sprouting capabilities of HBMEC in vitro after SB treatment [26,40]. Furthermore, SB is known to impair the development of VM, which has been shown to be a prognostic marker for GBM [25,70]. However, we only observed a tendency for decreased tumor vascularization after combined treatment. In comparison to our previous study, the antiangiogenic effect was less distinctive than when SB was administered alone [40]. The major difference between both set ups was the number of inoculated tumor cells, which was 5-fold higher in this study. This could have led to a different basis, as in this study, the morphological properties of the control tumors changed and were reflected in a 1.9-fold higher number of proliferating cells and only half the TAM numbers. 

Despite the relatively small effect of the combination therapy in our study, it is likely that other treatment protocols and longer observation periods could improve the treatment efficacy. For instance, cyclic TMZ protocols have led to a superior tumor volume reduction in comparable GL261 mouse models [71,72]. The treatment period in our study was restricted to only seven days due to the mini-osmotic pump for SB, since the efficacy of systemically administered SB has recently been shown to be lower [40]. In this regard, targeted approaches coupling SB with tumor-specific molecules (L19-SIP, F8-SIP) should be implemented to increase the antitumoral efficacy of systemic SB in order to be able to carry out survival studies [73,74]. Additionally, the value of sequential therapy regimens should be investigated [75], as the TMZ-induced upregulation of CXCR2 signaling and its ligands becomes more relevant over time [28,31]. Finally, the radiation therapy, which is part of the current standard therapy (Stupp protocol) in clinical routine, was not included in our experimental set-up [2]. However, SB225002 also has a radio-sensitizing effect [76]. Thus, we established an appropriate irradiation set-up to be able to implement irradiation in the combined treatment approach in the future studies [77]. 

### 3.5. Limitations

The major limitation of the patient-associated part was its retrospective design and selection bias with regard to the primary tumor evaluation as the second surgery for the recurrence was obligatory. However, this selection was necessary for the aimed direct matched primary and recurrent tumor evaluation as this was our focus. When comparing the treatment groups according to the TMZ amount, the lack of a TMZ-naïve main group must be criticized, but as the number of patients without any TMZ was very low, the suggested classification based on the amount of TMZ cycles was reasonable to gain first insights into the potential role of TMZ. 

The main limitations of our in vivo study were the small group size and the short treatment period as discussed above. Nevertheless, this set-up was suitable for providing initial findings on the tolerability and effectiveness of a combined treatment approach.

## 4. Materials and Methods

### 4.1. Human Specimens

**Figure 7:**
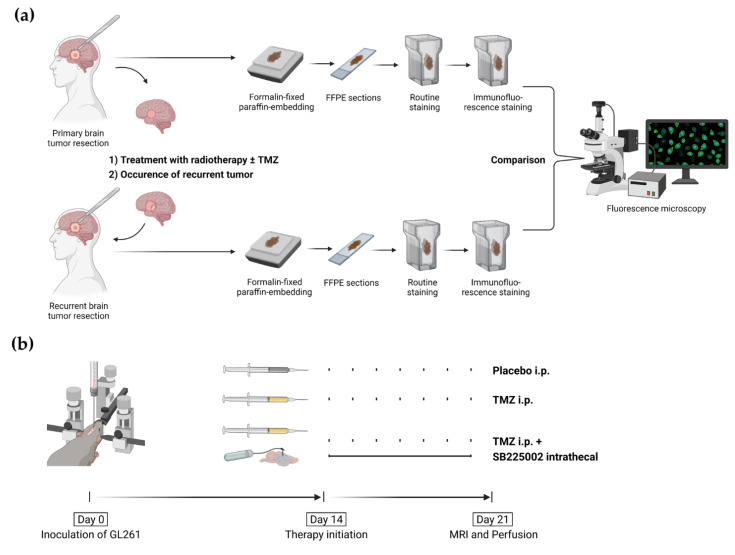
Methodical set-up. (**a**) GBM patients with primary and recurrent tumors were selected. Tumor tissue was formalin-fixed and embedded in paraffin (FFPE). FFPE sections were obtained and stained for routine parameters to confirm the diagnosis. FFPE sections were then stained for multiple additional markers and analyzed by fluorescence microscopy. (**b**) GL261 tumor cells were inoculated on day 0. Before the start of therapy, two weeks after tumor cell inoculation, first MRI was conducted to verify tumor growth. On day 21, a second MRI was conducted to assess tumor volume and animals were subsequentially perfused for brain harvesting. Harvested brains were used for immunofluorescence and qRT-PCR analysis. This figure was created with BioRender.com (accessed on 12 July 2021).

We performed a search in our retrospective GBM patient pool to identify patients that were treated for primary and recurrent GBM with surgical resection. Approval of the Ethical Committee of Charité–Universitätsmedizin Berlin, Germany was received for the retrospective assessment of patient data and residual tumor material (application number: EA1/045/18). All analyses were carried out based on the well-defined guidelines of good scientific practice working with patient material. According to our inclusion criteria, all GBM patients that underwent a resection for their primary and recurrent tumors between 2012 and 2017 at the Department of Neurosurgery, Charité–Universitätsmedizin Berlin, Germany, were screened. Furthermore, tumors had to be classified as GBM by two independent neuropathologists according to the latest WHO classification [54,78], using standard histological markers. A minimum of two months between the first and second surgery was set as an additional inclusion criterion to define it as recurrence after the primary tumor resection. Two patients had to be excluded from the analysis due to the lack of FFPE material. A total of 76 brain tissue samples of 38 patients with matched primary and recurrent GBM tumors were included in this study (Figure 7a). Clinical and routine diagnostic parameters, such as patient age, gender, progression-free survival, overall survival, MGMT-promotor methylation, IDH-1 mutations, and p53 accumulation, were assessed. Afterwards, detailed immunofluorescence stainings were performed to further compare primary and recurrent tumors (Figure 7a). 

The patients were further divided into two treatment groups, depending on the de facto received therapeutic regimens for their primary tumors. Patients that were treated by almost complete standard Stupp protocol for their primary GBM [2], consisting of primary tumor resection followed by radio-chemotherapy and six cycles of adjuvant chemotherapy, were classified as the “standard” group. To be able to provide a similar size of the groups to allow comparisons, ≥4 adjuvant TMZ cycles were included in the standard group (TMZ^≥4^). Patients that received ≤3 cycles or temozolomide or none were classified as “not standard” (TMZ^≤3^). 

#### Immunofluorescence Staining of Human FFPE Sections

First, 4-µm-thick FFPE slices of 76 tumor samples were deparaffinized in xylol and rehydrated in a descending alcohol series. For optimal antigen retrieval, FFPE slices were pressure cooked in a citrate buffer for 15 min followed by washing steps with aqua bidest and PBS. After blocking with 0.5%, 1% casein, or 10% goat serum, respectively, the following primary antibodies were used: polyclonal goat anti-IBA1 (1:100, abcam, Cambridge, UK, ab5076), polyclonal rabbit anti-CXCL2 (1:100, BIO-RAD, Hercules, CA, USA, AHP773), monoclonal mouse anti-IL8 (1:100, R&D Systems, Minneapolis, MN, USA, MAB208-100), polyclonal rabbit anti-VEGF (1:100, abcam, Cambridge, UK, ab1316), polyclonal rabbit anti-CXCR2 (1:100, abcam, Cambridge, UK, ab14935), and polyclonal mouse anti-CD31 (1:25, R&D Systems, Minneapolis, MN, USA, BBA7). For the anti-IBA1, anti-CXCL2, and anti-IL8 staining, the Autofluorescence Eliminator Reagent (Millipore, Burlington, MA, USA) was applied according to the manufacturer´s instructions. Slices were incubated with primary antibodies for 2 h followed by several washing steps and treatment with respective secondary antibodies (Alexa Fluor^®^647 donkey anti-goat, 1:200; Cy^TM^3 donkey anti-rabbit, 1:200; rhodamine red conjugated donkey anti-mouse, 1:200; fluorescein conjugated donkey anti-rabbit, 1:200; all Jackson ImmunoResearch Europe Ltd., Ely, UK) for 1.5 h. All slides were covered with DAPI-containing mounting medium (Dianova, Hamburg, Germany) and sealed with cover slips. 

Images were acquired with a 20× magnifying objective using a fluorescence microscope (Zeiss, Axio Observer Z1, Zeiss MicroImaging GmbH, Jena, Germany). ImageJ 1.53c (available from: http://imagej.nih.gov/ij, accessed on 28 June 2020) was used to analyze images. In total, 5–22 images from each patient derived from throughout the whole available tumor area were analyzed. For the subgroup analysis of TAM infiltration, the mean of all patients was defined as the limit for patients with low infiltration (<520 cells/mm^2^). The group with >520 TAMs was further divided into high (520–1000 cells/mm^2^) and very high infiltration (>1000 cells/mm^2^). 

### 4.2. In Vivo Animal Model

#### 4.2.1. Tumor Cells

For tumor initiation, we used murine GL261 cells. Prior to inoculation, cells were cultured for three days in Dulbecco’s Modified Eagle Medium supplemented with 1% Streptomycin/Penicillin and 10% fetal bovine serum at 5% CO_2_ at 37 °C. The cells were harvested at around 70% confluence for the inoculation. 

#### 4.2.2. Animals and Set-Up

All experiments were conducted according to German Law for Animal Protection under the permission number G0221/17 controlled by LaGeSo Berlin. Additionally, ARRIVE Guidelines were followed. A syngeneic mouse model with GL261 glioblastoma cells was used. Animals (C57BL6N, female, 11 weeks, 20–24 g) were kept in 12 h light–dark cycle, fed and watered ad libitum. Animals were checked daily for neurological symptoms and changes in weight. Animals received intraperitoneally administered anesthesia in standard dosages (9 mg Ketamine-Hydrochloride + 1 mg Xylazine per 100 g) for tumor cell inoculation, mini-osmotic pump implantation, and perfusion. Additionally, mice received infection prophylaxis (Benzylpenicillin-Natrium, InfectoPharm^®^, Heppenheim, Germany) and analgesic protection (Paracetamol, B.Braun, Mesungen, Germany) during the procedures.

The experimental set up used in this study was based on the timeline described previously [40]. In brief, the investigation period lasted 21 days and started with tumor cell inoculation on day 0. A total of 1 × 10^5^ GL261 cells were stereotactically inoculated into the right striatum (2 mm lateral, 1 mm anterior, and 3 mm deep to the Bregma) using a 1 µL Hamilton syringe. After verification of tumor size by MRI, the treatments were initiated on day 14. After a treatment period of seven days, MRI was conducted again to analyze the tumor growth before sacrificing animals for further analyses (Figure 7b). 

#### 4.2.3. Treatments

SB was administered intrathecally as recently described [40]. In short, a mini-osmotic pump (model 2002/2001; ALZET, DURECT Corporation, Cupertino, CA, USA) was prepared and filled with the small molecule CXCR2 Antagonist SB225002 (Tocris, Bristol, UK) according to the manufacturer’s protocol prior to implantation on day 14. The intraventricular catheter was placed 0.8 mm laterally of the bregma into the lateral ventricle contralateral to the tumor site and a subcutaneous pocket was prepared for mini-osmotic pump reservoir placement. Treatments started immediately with implantation of a mini-osmotic pump on day 14. SB was administered continuously with a rate of 1 µL per hour at a dosage of 30 µg per day.

The intraperitoneal administration of TMZ (TEMODAL^®^, MSD, Kenilworth, NJ, USA) was conducted daily with a weight-adapted dosage of 60 µg/g/d. The control group received Aqua ad iniectabilia (B.Braun, Mesungen, Germany) intraperitoneally.

#### 4.2.4. MRI

Tumor volume was measured using a 7 Tesla small animal MRI (Pharmascan 70/16AS, Bruker BioSpin, Ettlingen, Germany and Paravision 5.1 software) and a 20 mm inner diameter quadrature volume resonator (Rapid Biomedical, Rimpar, Germany). Mice received inhalation anesthesia using 1.5–2.0% isoflurane (cp-pharma, Burgdorf, Germany) in a mixture of oxygen and nitrous oxide (30%/70%) and were placed on a heating plate to maintain body temperature. For continuous monitoring during MRI, respiratory frequency was measured. T1-weighted sequence (TR/TE = 800/10.6 ms, RARE factor 2, 4 averages) with contrast agent (Gadolinium, Magnevist^®^, Bayer AG, Leverkusen, Germany) and T2-weighted sequence (TR/TE = 4200/36 ms, RARE factor 8, 4 averages) was conducted. Volumes were measured by Analyze 10.0 (Analyze 10.0, AnalyzeDirect, Inc., Overland Park, KS, USA). 

#### 4.2.5. Tissue Harvesting and Preparation for Analysis

Prior to brain harvesting on day 21, animals were anaesthetized and then perfused intracardially with Paraformaldehyde (PFA) 4% in PBS (phosphate-buffered saline) or sterile PBS alone. To prepare immunofluorescence staining, brains were post-fixed in 4% PFA for 24 h following by sucrose dehydration before freezing and storing at −80 °C. In preparation for PCR analysis, PBS-perfused brains were processed under the microscope to resect tumor tissue as well as contralateral control tissue microsurgical. Both were shock frozen in liquid nitrogen and stored at −80 °C until proceeding with tissue homogenization for RNA extraction.

#### 4.2.6. Immunofluorescence Staining of Mouse Brain Sections

Frozen brains were cut into 10 µm sections at a temperature of −29 °C (Thermo Scientific (Microm HM 560), Waltham, MA, USA) before staining for proliferation, apoptosis, vasculature, and TAM accumulation according to standard protocols. Apoptosis was stained according to the manufacturer’s recommendations using a TUNEL Kit (TUNEL, ApopTag red In situ kit (S7165), Merck Millipore, Darmstadt, Germany) followed by proliferation (rabbit anti-KI67, Thermo Fisher Scientific (RM-9106-S1), Waltham, MA, USA; 1:200, 16 h, 4 °C) staining in a combined protocol. The other primary antibodies were used as listed: TAMs (rabbit anti-IBA1, Wako Chemicals (019-19741), Richmond, VA, USA; 1:200); endothelial cells (rat anti-CD31, BD Pharmingen (550274); 1:50, 16 h, 4 °C); CXCL2 (goat, R&D Systems (AF-452-NA), Minneapolis, MN, USA; 1:20, 16 h, 4 °C), and CXCR2 (rabbit, abcam (ab14935), Cambridge, UK; 1:200, 48 h, 4 °C).

All secondary antibodies (Alexa Fluor^®^ 647 donkey anti rabbit, Jackson ImmunoResearch (711-605152); CyTM3 donkey anti-rat, Jackson ImmunoResearch (712-165-153); Alexa Fluor^®^ 647 donkey anti-goat, Jackson ImmunoResearch (705-605-147); West Grove, PA, USA) were used at a concentration of 1:200 for 90–120 min at room temperature solved in Casein 0.5% in PBS. The final step of all protocols included staining of nuclei with DAPI (Dianova, Hamburg, Germany) and subsequently covering slides for immunofluorescence microscopy. To obtain a comprehensive overview of the tumor area, images were taken of at least three sections of different regions per animal and per staining. Images were acquired as earlier described with a 20× magnifying objective using a fluorescence microscope and ImageJ 1.53c was used to analyze images. 

#### 4.2.7. RNA Isolation and Quantitative Real-Time PCR 

RNA from tumor-bearing mice was isolated from homogenized tumor tissue as well as contralateral parenchyma of the control group using a RNeasy Kit (QIAGEN, Hilden, Germany) according to the manufacturer’s protocol. RNA concentration and quality was measured with a plate photometer (Infinite M200, Tecan, Männedorf, Switzerland) prior to eradication of genomic DNA contamination. cDNA synthesis of tumor tissue as well as contralateral parenchyma was carried out with 500 ng RNA using the PrimeScript^TM^RT reagent Kit with gDNA Eraser (TaKaRa, Cat. #RR047A, Shiga, Japan) according to the manufacturer’s instructions. The cDNA quantity was measured by photometry. Quantitative Real-time-PCRs (qRT-PCRs) were executed for *Cxcr2*, *Cxcl2*, *Cxcr1*, *Bax*, *Bcl-2*, *Vegf*, *Vegfr1*, and *Vegfr2* using triplicates in a 10 µL reaction volume and the TB Green^TM^ Premix Ex Taq^TM^ Kit (TaKaRa, Shiga, Japan). *18S* was used as the reference gene. Primer sequences can be found in the Supplementary Materials Table S3. qRT-PCRs were performed with the Quant Studio 6 Flex System (Thermo Scientific, Waltham, MA, USA). Target expression levels were normalized to *18S* mRNA. The relative quantification method (^ΔΔ^Ct) was used for analyses.

### 4.3. Statistical Analysis

Statistical analyses were performed using GraphPad Prism Software (v9.1.1, San Diego, CA, USA) and IBM SPSS Statistics (Version 26.0 Armonk, NY, USA, IBM Corp.). Data was presented as mean ± standard deviation (SD) or/and median with (1st and 3rd quartile). The mean of the mean per group was used for the immunofluorescence analyses. Primary and recurrent tumors were compared by two-tailed unpaired Student´s *t*-test, while a two-tailed paired Student´s *t*-test was performed for the analysis of primary and recurrent tumors within the same treatment groups, and mean differences and the 95% confidence interval of these tests are presented as indicated. Differences in murine treatment groups were first analyzed by one-way ANOVA to detect if any significant changes existed amongst the groups. If there was a significance in ANOVA analysis, single comparisons with Bonferroni correction were applied and significant results are reported. Due to the exploratory character of this study, *p*-values and confidence intervals were not adjusted for multiplicity (except pairwise comparisons after ANOVA). The overall survival and progression-free survival were investigated using Kaplan–Meier analysis followed by log-rank-test to compare groups. In order to assess the relevance of multiple factors, univariable and multiple analyses were performed using Cox regression analyses. Multiple analysis was carried out for all variables with *p* ≤ 0.2 in the univariable analysis. Pearson’s correlation coefficient was used to determine the association between the expression levels of different proteins. A *p* value ≤ 0.05 was considered as significant. For better readability, we only stated *p* values ≤ 0.055 in the text to highlight the significant or almost significant results.

## 5. Conclusions

To our knowledge, this is one of the largest series comparing matched GBM tumors. We identified TAM infiltration as a negative predictor of OS in GBM. Our in-depth analysis of matched GBM tumors showed that primary tumors were highly infiltrated by TAMs in comparison to recurrent tumors and that a very high TAM infiltration drives primary tumor progression. Interestingly, TMZ treatment partially altered vascular parameters, IL8 and CXCL2 expression in GBM patients. In order to fully elucidate the in vivo effect of TMZ with a focus on TAMs and the important signaling pathways in GBM patients, further prospective studies are warranted.

Our novel therapeutic approach combining TMZ with CXCR2 antagonization in vivo was well-tolerated and led to decreased tumor volumes despite the short treatment period of only seven days. Thus, this study provides promising first insights into this combination treatment. The full efficacy of this combinational approach should be investigated in further preclinical studies with a longer observation period.

## Figures and Tables

**Table 1:** Patients’ characteristics.

**Patient Features**		***n***	
**female**		13 * (34%)	
**male**		25 * (66%)	
**mean age at diagnosis in years**		59 ± 13	
**median age at diagnosis in years**		61 (21–82)	
**Treatment**			
**primary tumor resection**		38 * (100%)	
**tmz cycles**	≥4	18 * (47%)	
	1–3	8 * (21%)	
	0	12 * (32%)	
**Survival**			
**PFS (months)**	mean	12 ± 9	
	median	9 (2–36)	
**OS (months)**	mean	19 ± 10	
	median	17 (2.4–42.5)	
**Histopathologic Features**			
**GBM samples**		76 (100%)	
**MGMT status**	unmethylated	22 (58%) *	
	methylated	16 (42%) *	
**IDH1 status**	mutated	1 (2.6%) *	
	wildtype	37 (97.4%) *	
**Ki-67 (mitotic index)**		pGBM	rGBM ^#^
	<10%	2 (5.3%)	7 (20%)
	10–30%	32 (84.2%)	26 (74.3%)
	>30%	4 (10.5%)	2 (5.7%)
**p53 accumulation**	positive	34 (89.5%)	26 (81.25%)
	negative	4 (10.5%)	6 (18.75%)

pGBM = primary GBM, rGBM = recurrent GBM, PFS = progression-free survival, OS = overall survival; * matched patients, ^#^ data was not available for all patients.

**Table 2:** Cox regression analysis of the overall survival.

OS	Univariable Analysis			Multiple Analysis		
	*p*-Value	HR	95% CI	*p*-Value	HR	95% CI
**pIBA1**	0.02	1.01	1.00–1.03	0.02	1.01	1.00–1.03
**pVEGF**	0.57	1.07	0.85–1.33			
**pCXCL2**	0.60	1.04	0.85–1.22			
**pIL8**	0.57	0.80	0.37–1.73			
**pvessel count**	0.77	1.00	0.99–1.02			
**pvessel area**	0.45	0.00	0.00–1,873,571.60			
**pCXCR2^+^ vessels**	0.72	1.00	0.99–1.02			
**pCXCR2^+^ vessel area**	0.12	0.00	0.00–8874 × 10^22^	0.04	0.00	0.00–0.001
**TMZ cycles**	0.20	0.94	0.86–1.03	0.07	0.86	0.73–1.01
**MGMT methylation**	0.30	0.99	0.96–1.01			

p = primary tumor; TMZ = temozolomide; HR = Hazard Ratio; 95% CI = 95% confidence interval.

**Table 3:** Cox regression analysis of the progression-free survival.

PFS	Univariable Analysis			Multiple Analysis		
	*p*-Value	HR	95% CI	*p*-Value	HR	95% CI
**pIBA1**	0.60	1.00	0.99–1.00			
**pVEGF**	0.35	0.90	0.73–1.12			
**pCXCL2**	0.29	1.08	0.93–1.26			
**pIL8**	0.60	0.82	0.39–1.72			
**pvessel count**	0.48	1.01	0.99–1.02			
**pvessel area**	0.49	1114.81	0.00–6.63 × 10^11^			
**pCXCR2^+^ vessels**	0.25	0.99	0.98–1.01			
**pCXCR2^+^ vessel area**	0.32	0.00	0.00–4.34 × 10^41^			
**TMZ cycles**	0.01	0.86	0.77–0.96	0.02	0.87	0.78–0.98
**MGMT methylation**	0.04	0.97	0.94–0.99	0.09	0.98	0.95–1.00

p = primary tumor; TMZ = temozolomide; HR = Hazard Ratio; 95% CI = 95% confidence interval.

## Data Availability

The datasets used and analyzed during the current study are available from the corresponding author (GA) upon request.

## Supplementary Materials

The following are available online at https://www.mdpi.com/article/10.3390/ijms222011180/s1.
