# Supplementary material for: Tumor-Associated Microglia/Macrophages as a Predictor for Survival in Glioblastoma and Temozolomide-Induced Changes in CXCR2 Signaling with New Resistance Overcoming Strategy by Combination Therapy
Source: Int J Mol Sci. 2021 Oct 16;22(20):11180. doi: 10.3390/ijms222011180 (PMC8538679; doi:10.3390/ijms222011180)
Supplement: Supplementary file 1 [file ijms-22-11180-s001.zip › ijms-1370060-supplementary.pdf]

International Journal of
Molecular Sciences

MDPI

Supplementary Materials

Supplementary Tables

Table S1. **P**atients' characteristics stratified in groups.

Study Cohort: *n* = 38	*n*	%	*p*-value
**Patient Characteristics**			
gender (f/m)	13/25	34/66	
Standard	8/10	44/56	0.22
Not Standard	5/20	25/75	
age at diagnosis of pGBM in years (MW ± SD)	58.7 ± 12.9		
Standard	56.8 ± 12.3		0.41
Not Standard	60.3 ± 13.5		
age at diagnosis of rGBM in years (MW ± SD)	59.6 ± 12.8		
Standard	58.2 ± 12.3		0.50
Not Standard	61.0 ± 13.5		
**Histopathological Characteristics of Primary GBM**			
MGMT (methylated ≥10%/unmethylated <10%)	16/22	42/58	
Standard	10/8	55/45	0.22
Not Standard	6/20	30/70	
IDH1/2-Status (wt/mut)	37/1	97.4/2.6	
Standard	17/1	94.5/5.5	0.30
Not Standard	20/0	100/0	
TP53 (+/-)	34/4	89.5/10.5	
Standard	18/0	100/0	0.05
Not Standard	16/4	80/20	
IBA1 cells/mm^2 (MW ± SD)	520 ± 330		
Standard	518 ± 309		0.95
Not Standard	523 ± 356		
VEGF expression (MW ± SD)	2.2 ± 2.1		
Standard	2.4 ± 1.7		0.56
Not Standard	2.0 ± 2.4		
CXCL2 expression (MW ± SD)	3.1 ± 2.1		
Standard	2.6 ± 2.3		0.27
Not Standard	3.4 ± 1.8		
IL8 expression	43%		
Standard	44.4%		0.33
Not Standard	42.1%		

f = female; m = male; wt = wild type; mut = mutant, + = mutant; - = not mutant, pGBM = primary GBM, rGBM = recurrent GBM. Supplementary table 1 contains the clinical information of all patients and the subgroup analysis of patients receiving the standard therapy according to Stupp´s protocol [1] with ≥ 4 adjuvant cycles of TMZ (Standard) and patients receiving ≤3 adjuvant cycles of TMZ (not standard).

Table S2. Cox-regression analysis excluding CXCR2 negative patients (n = 18).

OS	univariate analysis			multiple analysis		
	p-value	HR	95% CI	*p*-value	HR	95% CI
**pIBA1**	0.02	1.00	1.00 - 1.00	0.03	1.01	1.00 – 1.03
**pCXCR2⁺vessel area**	0.15	0.00	0.00 - 1457 x 10^{30}	0.08	0.00	0.00 - 2078 x 10^{10}
**TMZ cycles**	0.20	0.94	0.86 - 1.03	0.26	0.90	0.74 - 1.08

p = primary tumor; TMZ = temozolomide; HR = Hazard Ratio; 95% CI = 95% confidence interval.

Table S3. Primer sequences for qRT-PCR

Gene	Primer Orientation	Sequence 5′ → 3′
18s [2]	forward	AACCCGTTGAACCCCATT
	reverse	CCATCCAATCGGTAGTAGCG
Bax [3]	forward	TGAAGACAGGGGCCTTTTTG
	reverse	AATTCGCCGGAGACACTCG
Bcl2 [4]	forward	GTCGCTACCGTCGTGACTTC
	reverse	CAGACATGCACCTACCCAGC
Cxcl2 [2]	forward	CGCTGTCAATGCCTGAAG
	reverse	GGCGTCACACTCAAGCTCT
Cxcr2 [5]	forward	AGCAAACACCTCTACTACCCTCTA
	reverse	GGGCTGCATCAATTCAAATACCA
Cxcr1 [6]	forward	AATCTGTTGTGGCTTCACCCA
	reverse	GCTATCTTCCGCCAGGCATAT
Vegf [2]	forward	GAAGAAGAGGCCTGGTAATGG
	reverse	AAGCCACTCACACACACAGC
Vegfr1 [2]	forward	CTAATGACGATGGCAACAGG
	reverse	GCTAGCATGCTCTGCTCTCC
Vegfr2 [2]	forward	TGGAGGAAGAGGAAGTGTGC
	reverse	TCAGCTCTTCTGATGCAAGG

Primer sequences were purchased from TIB MOLBIOL, Berlin, Germany.

Supplementary Figures

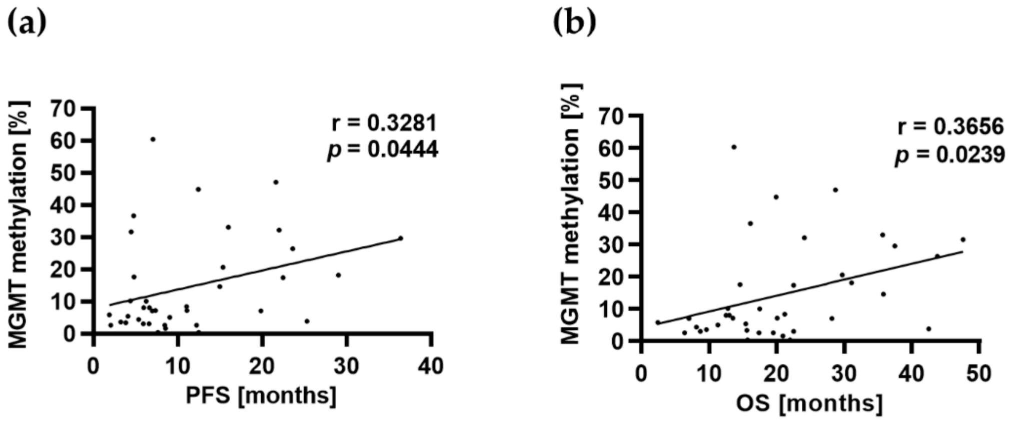

Figure S1. Pearson correlation analyses of the MGMT-status with PFS and OS of GBM patients. Statistical evaluation of clinical data with pearson correlation analyses. **(a,b)** MGMT methylation significantly correlates with a prolonged PFS and OS. Correlation coefficient according to pearson´s correlation analysis, linear regression analyses were performed. r and significant p values are indicated; p ≤ 0.05 was considered statistically significant, n = 38

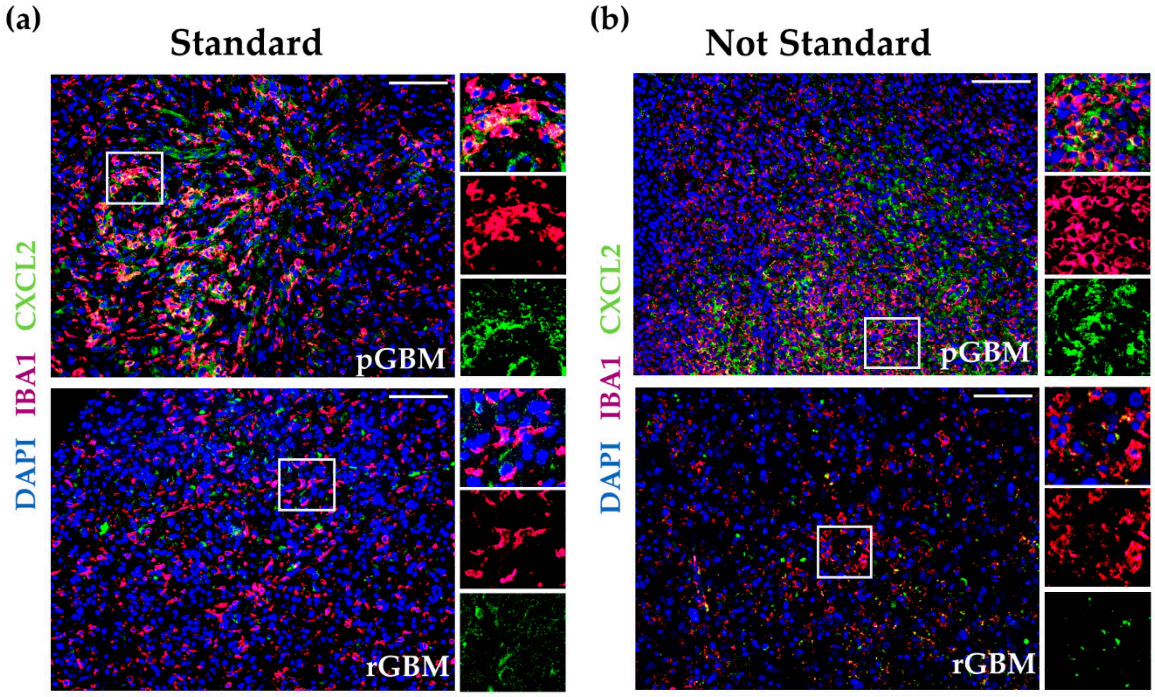

Figure S2. CXCL2 expressing TAMs. (a, b) Representative immunofluorescence staining of TAMs (IBA1) in magenta, CXCL2 in green and cell nuclei (DAPI) in blue of the standard and not standard group in primary and recurrent tumors of GBM patients, scale bar 100 μm.

References

1. Stupp, R.; Mason, W.P.; van den Bent, M.J.; Weller, M.; Fisher, B.; Taphoorn, M.J.; Belanger, K.; Brandes, A.A.; Marosi, C.; Bogdahn, U.; et al. Radiotherapy plus concomitant and adjuvant temozolomide for glioblastoma. *N Engl J Med* **2005**, *352*, 987-996, doi:10.1056/NEJMoa043330.
2. Brandenburg, S.; Muller, A.; Turkowski, K.; Radev, Y.T.; Rot, S.; Schmidt, C.; Bungert, A.D.; Acker, G.; Schorr, A.; Hippe, A.; et al. Resident microglia rather than peripheral macrophages promote vascularization in brain tumors and are source of alternative pro-angiogenic factors. *Acta Neuropathol* **2016**, *131*, 365-378, doi:10.1007/s00401-015-1529-6.
3. Tao, J.; Shen, X.; Ai, Y.; Han, X. Tea polyphenols protect against ischemia/reperfusion-induced liver injury in mice through anti-oxidative and anti-apoptotic properties. *Exp Ther Med* **2016**, *12*, 3433-3439, doi:10.3892/etm.2016.3789.
4. Huang, Q.; Xiong, H.; Yang, H.; Ou, Y.; Zhang, Z.; Chen, S.; Ye, Y.; Zheng, Y. Differential Expression of Bcl-2 in the Cochlea and Auditory Cortex of a Mouse Model of Age-Related Hearing Loss. *Audiology & neuro-otology* **2016**, *21*, 326-332, doi:10.1159/000450937.
5. Reutershan, J. Critical role of endothelial CXCR2 in LPS-induced neutrophil migration into the lung. *Journal of Clinical Investigation* **2006**, *116*, 695-702, doi:10.1172/jci27009.
6. Fan, X.; Patera, A.C.; Pong-Kennedy, A.; Deno, G.; Gonsiorek, W.; Manfra, D.J.; Vassileva, G.; Zeng, M.; Jackson, C.; Sullivan, L.; et al. Murine CXCR1 Is a Functional Receptor for GCP-2/CXCL6 and Interleukin-8/CXCL8. *Journal of Biological Chemistry* **2007**, *282*, 11658-11666, doi:10.1074/jbc.m607705200.
